# Influence of Methylenetetrahydrofolate Reductase *C677T*, *A1298C*, and *G80A* Polymorphisms on the Survival of Pediatric Patients with Acute Lymphoblastic Leukemia

**DOI:** 10.1155/2012/292043

**Published:** 2012-10-17

**Authors:** Dayse Maria Vasconcelos de Deus, Elker Lene Santos de Lima, Rafaela Maria Seabra Silva, Edinalva Pereira Leite, Maria Tereza Cartaxo Muniz

**Affiliations:** ^1^Pediatric Hematology Oncology Center (CEONHPE), UPE, Avenida Agamenon Magalhães, Bairro de Santo Amaro, 50100-010 Recife, PE, Brazil; ^2^Department of Tropical Medicine, Federal University of Pernambuco (UFPE), Avenida Moraes Rego, 1235 University City, 50670-901 Recife, PE, Brazil; ^3^Biological Sciences Institute, University of Pernambuco (UPE), Avenida Agamenon Magalhães, Bairro de Santo Amaro, 50100-010 Recife, PE, Brazil; ^4^University of Pernambuco (UPE), Avenida Agamenon Magalhães, Bairro de Santo Amaro, 50100-010 Recife, PE, Brazil

## Abstract

The influence of genic polymorphisms involved in metabolism of chemotherapeutic agents as the methotrexate (MTX) has been studied mainly in acute lymphoblastic leukemia (ALL) of childhood. Advances in treatment may be attributed to identification of prognostic factors added to chemotherapy protocol. The aim of this study was to analyze the association of the *C677T*, *A1298C*, and *G80A* polymorphisms on *MTHFR* gene and on the overall survival of pediatric patients (*n* = 126) with lymphoblastic leukemia treated with MTX according to the Brazilian protocol in 187 months. The *C677T* and *G80A* polymorphisms were genotyped by PCR-RFLP and *A1298C* polymorphism by allele-specific PCR. We observed that ALL patients presented rate (dead/alive) of 0.36 for the *677CC* genotype, corresponding also to lower overall survival (*P* = 0.0013); on the other hand, the *677TT* genotype showed a better survival (98%). Thus, we believe that patients with *80AA* genotype presented a small reduction in MTX plasma level, suggesting that ALL children, carrying the *80AA* genotype, showed a high toxicity to MTX (*P* < 0.0001).

## 1. Introduction

Leukemia is the most common childhood cancer. Recently the influence of polymorphisms in different genes is involved on the metabolism of chemotherapeutic agents and it has been studied especially in childhood acute lymphoblastic leukemia (ALL) [1]. Despite actual chemotherapy protocols cure almost 80% of pediatric patients with ALL, the majority of adult patients still die from this disease. Advances in cure rates in children could be attributable to identification of prognostic features together with the intensified chemotherapy and improved supportive therapy [2]. Genetic polymorphisms in patients with ALL can alter drug-metabolizing enzymes, transporters, and targets; therefore, they can influence both efficacy and toxicity of chemotherapeutic agents. Actually this type of genetic polymorphisms is not used in a specific treatment; however, they could be responsible for an altered sensitivity of leukemic cells to drugs [3]. The pharmacological pathway of MTX is useful to identify genes and polymorphisms that influence the response to chemotherapy for ALL. An important enzyme in the folate/methotrexate metabolism pathway is 5,10-methylenetetrahydrofolate reductase (*MTHFR*), which catalyzes the conversion of 5,10-methylenetetrahydrofolate to 5-methyltetrahydrofolate in the folic acid cycle [3]. The *MTHFR* plays an important role in the folate metabolism and differences in its activity due to these two genic variants might modify the modulation of therapeutic response to antifolate chemotherapeutic agents. The frequencies of the *C677T* and *A1298G* allelic variants vary by ethnicity. In Europe, 8–20% of the Caucasian population is homozygous for the *677T* allele and almost 40% is heterozygous [4]. The human reduced folate carrier (*RFCh*) is expressed in all cells and it is characterized as primarily responsible for the transport of folate and antifolate chemotherapeutic drugs, such as methotrexate (MTX), pemetrexed (Alimta), and raltitrexed (Tomudex) in mammalian cells, even when multiple other systems of assimilation are present [5]. However, the importance of physiology and pharmacology of *G80A* (*RFCh*) polymorphism remains unclear, although clinical and epidemiological findings have shown controversy [6]. In this study, we analyzed the association between the *C677T*, *A1298C*, and *G80A* polymorphisms and overall survival of Brazilian children with ALL submitted to treatment according to the Brazilian Group for Treatment of Lymphoblastic Leukemia in Childhood (GBTLI-99).

## 2. Materials and Methods

### 2.1. Patients and Samples

One hundred twenty-six children with acute lymphoblastic leukemia aged 0 ≤ 18 years were studied. They were enrolled in the Pediatric Hematology Oncology Center, Hospital Oswaldo Cruz (HUOC), Recife, Brazil, from 2003 to 2011. We evaluated 126 patients for *C677T* polymorphism and 118 patients for *A1298C* and *G80A* polymorphisms regarding to overall survival, and they presented clinical and laboratory diagnosis for acute leukemia using the GBTLI-99 treatment protocol [7]. The patient samples were collected from bone marrow puncture in the posterior iliac crest, according to the ethics committee of HUOC. The DNA samples were obtained by salting-out method (1988) [8].

### 2.2. Genotyping

The *C677T* polymorphism was genotyped by PCR-RFLP method as previously described [9]. The primers used for *MTHFR* genotyping of the *C677T* polymorphism were (forward) 5′-TGA AGG AGA AGG TGT CTG CGG GA-3′ and (reverse) 5′-AGG ACG GTG CGG TGA GAG TG-3′. The cycling: 1 cycle of 95°C/6 min, 40 cycles of 95°C/60 s, 62,5°C/90 s, and 72°C/60 s and 1 cycle of 72°C/7 min. Each PCR reaction of 24 *μ*L contains the components: 2.5 *μ*L Buffer (10x), 1 *μ*L MgCl_2_ (50 mM), 1 U Taq polymerase (5 U/*μ*L), 2 *μ*L dNTP (200 *μ*M), 1.5 *μ*L primer (5 pmol/*μ*L). The *677C* → T base pair substitution creates a *Hinf1* restriction site. The PCR product (198 bp) of *C677T* was digested for 48 hours at 37°C using *Hinf1* and analyzed on agarose gel 3% with ethidium bromide (0.4 mg/mL) in electrophoresis. Digestion of PCR product with *Hinf1 *showed fragments of 175 bp and 23 bp for the TT genotype, 198 bp, 175 bp, and 23 bp for the CT genotype.

The *A1298C* polymorphism was genotyped by adapted allele specific PCR [10], were used (allele A) forward 5′-GGA GCT GAC CAG TGA AGA-3′ and reverse 5′-TGT GAC CAT TCC GGT TTG-3′; (allele C) forward 5′-CTT TGG GGA GCT GAA GGA-3′ and reverse 5′-AAG ACT TCA AAG ACA CTT G-3′. The cycling: 1 cycle of 94°C/2 min, 30 cycles of 95°C/30 s, 58°C/30 s, and 72°C/50 s and 1 cycle of 72°C/5 min. Each PCR, for 23 *μ*L reaction contains the components: 2.3 *μ*L Buffer (10x), 0.75 *μ*L MgCl_2_ (50 mM), 1.5 U Taq polymerase (5 U/*μ*L), 2 *μ*L dNTP (200 *μ*M), and 2 *μ*L each primer (5 pmol/*μ*L). The amplified products were analyzed using agarose gel 4% with ethidium bromide (0.4 mg/mL) by electrophoresis.

The *G80A* polymorphism was genotyped by PCR-RFLP using the primers and enzyme HhaI according to Chango et al. [11].

### 2.3. Evaluation of Toxicity and MTX Plasma Concentrations

The toxicity was assessed by toxicity scale for blood, liver, and kidney in accordance with the National Cancer Institute-Common Toxicity Criteria (NCI-CTC), version 2.0. The MTX serum concentrations were evaluated in 24 hours and 48 hours during the maintenance phase, using the *Methotrexate II* kit (Abbott Laboratories) and the automatic analyzer of fluorescence—*FLX TDX* (Labclinics).

### 2.4. Statistical Analysis

The associations between categorical variables were performed using *χ* ^2^ test. The overall survival analysis was performed using the patient followup according to the statistical models in conjunction with *Kaplan Meier Log Rank* (*Mantel Cox*) to assess the risk of death in 15.58 years (187 months) time. The serum MTX concentrations were analyzed by *Kuskal Wallis* test. All results with *P* value <0.05 were statistically significant. For this analysis we used the statistical programs *BioEstat 5.0* and *GraphPad Prism 5.0*.

## 3. Results

The median age was 9 years; according to gender, the distribution of polymorphisms was similar, and except for the *A1298C* polymorphism, the *G80A* and *C677T* polymorphisms were in *Hardy-Weinberg* equilibrium. Regarding the rate (dead/alive), we observed that among the polymorphisms, the *677CC*, *1298AC*, and *80AA* genotypes showed the higher death proportion (Table 1).

**Figure 1:**
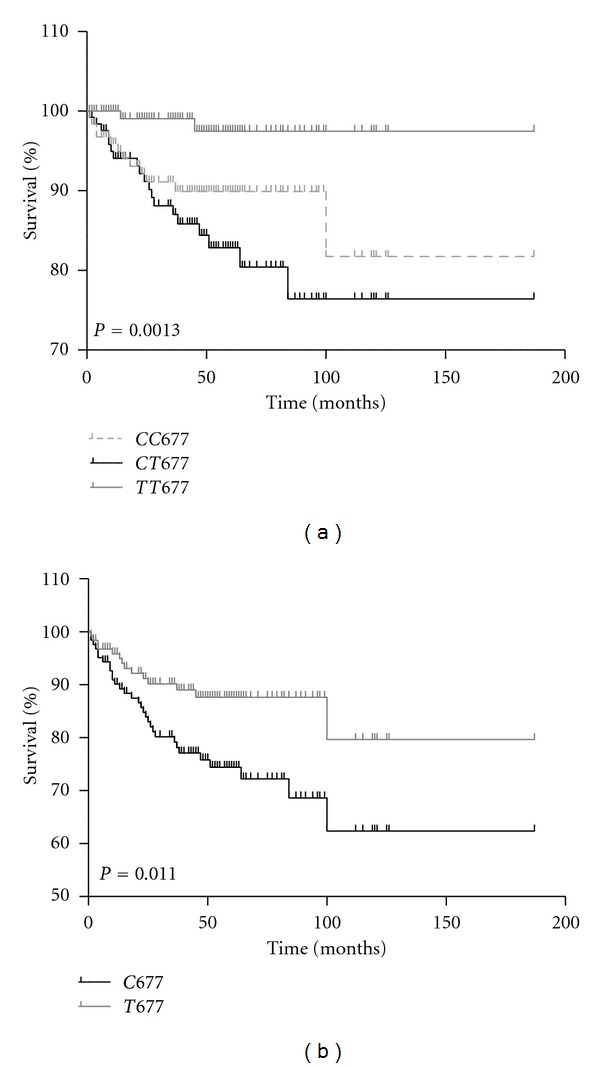
Overall survival curve of ALL patients with genotype of the *C677T* polymorphism. (a) The survival of patients who carry the *677CC* genotype is significantly lower than the survival of patients with the *677TT* or *677CT* genotypes and (b) the survival of patients who carry the variant *677C* allele is significantly lower than the survival of patients with the* 677T* allele.

The *C677T* polymorphism showed a better overall survival for the *677TT* genotype than *677CC* and *677CT* genotypes in ALL, due to allele C. The survival of ALL patients with the *677TT* genotype was about 98%, while for the *677CC* genotype was 77%. The *677C* allele favored the survival in 62%, while the *677T* allele was 80% (Figure 1).

The *A1298C* polymorphism showed a better overall survival for the *1298CC* genotype, showing a followup of 93%, while the AC genotype was 80% and AA was 85%, respectively. The survival of ALL patients with the A allele was about 68%, while that of patients with *1298C* allele was 74% (data not shown).

Patients with *80GA* genotype showed a better survival, while *80GG* genotype patients showed worse survival up to 80 months and the survival analyses related to the allele were observed when the curves differ to 80 months (data not shown).

We analyzed the toxicity in only 18 patients. We observed that, according to NCI-CTC, the patients with *80AA* genotype showed blood toxicity of grades 1 and 2, hepatic toxicity of grades 1 and 3, and no renal toxicity; patients with *80AG* genotype showed blood toxicity of grades 1, 2, and 3, liver toxicity of grades 2 and 3, and no renal toxicity; patients with *80GG* genotype presented just blood toxicity of grade 3 and no liver and kidney toxicities.

**Figure 2:**
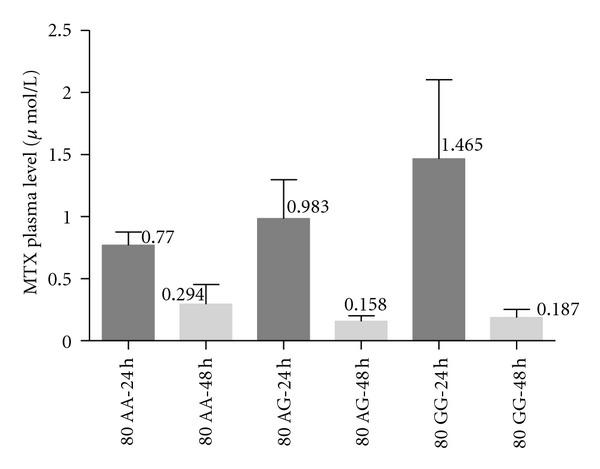
Frequency of MTX plasma levels of methotrexate in patients with genotype of the *G80A* polymorphism at 24 h and 48 h of clearance, by *Kruskal Wallis* test (*P* < 0.0001).

On the other hand, we report that the plasma levels of MTX in patients with *80AA* genotype, in the first 24 hours, showed lower MTX plasma concentrations (mean 0.77 *μ*mol/L; range 0.12–1.84; SD ± 0.438), while *80GG* genotype showed higher MTX plasma concentrations (mean 1.46 *μ*mol/L; range 0.36–5.45; SD ± 1.80); the *80GA* genotype had mean of 0.98 *μ*mol/L (range 0.31–5.12; SD ± 1.32). We did not observe large difference between *G80A *(*RFCh*) polymorphism and the MTX plasma level in 48 hours (*80AA* genotype: mean 0.30 *μ*mol/L; range 0.02–2.6; SD ± 0.651 ∣ *80GA* genotype: mean 0.158 *μ*mol/L; range 0.08–0.43; SD ± 0.173 ∣ *80GG* genotype: mean 0.18 *μ*mol/L; range 0.07–0.55; SD ± 0.171), (Figure 2).

## 4. Discussion

In these last years it has been extensively debated the influence of* C677T* and *A1298C MTHFR* polymorphisms on hematological malignancies. Particularly, the roles of both polymorphisms on the susceptibility of the development of ALL have been broadly discussed [4]. Nowadays, it is very important to analyze the pathogenesis of the disease and, consequently, the risk stratification for a different genotypic profile population. De Jonge et al. [12] analyzed 245 dutch children and found that the T allele decreases the risk of leukemia in these patients according to the toxicity in course of chemotherapy with methotrexate (MTX), and, more recently, on the clinical response to chemotherapy [13], which can vary in populations, because leukemia is a multifactorial disease. In our country, the folic acid intake during pregnancy is impaired [14, 15], and this deficiency is associated with changes in the pattern of DNA methylation, a significant elevation of plasma homocysteine, as well as, a significant decline in plasma levels of folate (5-methyl-THF) [9] and consequently the susceptibility to cancer.

A meta-analysis study concluded that *MTHFR 677TT* reduces the risk of death in adult with ALL, but not in children and the *MTHFR 1298A* > *C* polymorphism did not influence the evolution of ALL susceptibility in childhood or adulthood [16]. Semsei et al. [17] found high protection in boys carrying *677CT* and *1289AA* genotypes and less protection in girls with *1298AC* and *677CC* genotypes. However, the results are conflicting; some studies report protective effects for *MTHFR 677TT* [12, 18, 19] and *1298CC* [18, 19], whereas others have yielded relatively little or no evidence of effect least for position *677* of *MTHFR* gene [16, 20]. There are several possible reasons for these inconsistencies, one of which relates to small population size of most previous studies.

Our results demonstrate the importance of analyzing the overall survival, estimating a successful treatment with MTX during chemotherapy for different polymorphisms types, mainly because the* 677C *allele influences the disease risk. In our country it is evaluated only by odds ratio in acute lymphoblastic leukemia [18, 21]. Our study was the first to evaluate the overall survival for 15.58 years in children with ALL, according to the treatment GBTLI-99 protocol.

This study demonstrated that pediatric patients with *677TT* genotype had a better overall survival than the patients with *677CC* genotype for the *MTHFR* gene. On the other hand, it was observed in those children who had high death frequency, carrying *1298AC* and *677CC* genotypes, although this result has been not statistically significant.

Over recent years, several studies have investigated the relationship between *MTHFR* gene polymorphisms and toxicity during the therapy with methotrexate in childhood ALL [22–25]. Some studies did not find significant associations between the *677T* allele and toxicity [13, 23, 26, 27], although one study reported a small rate of toxicity episodes among patients carrying the *677T* allele [22]. On the other hand, a study showed that individuals with *677T* allele had to interrupt the treatment with methotrexate most often, suggesting that the *677T *allele serves as a toxicity predictor during the chemotherapy maintenance. These different results are probably a ttributable to several factors, such as the methotrexate/dose, treatment protocol, ethnic background, and number of patients analyzed [21, 24, 25].

In our protocol (GBTLI 99), the patients were treated with 2 g/m^2^/dose of methotrexate after 8 weeks of the induction phase, and it is the highest MTX dose given during the treatment for pediatric patients with ALL [8]. Kotnik et al. [28] evaluated different protocols (BFM95, BFM90, BFM86, BFM2002, BFM90NHL) of treatment to pediatric patients, which it was administered high doses of methotrexate (5 g/m^2^) and after analyzed the *C677T *and *A1298C* polymorphisms (also other genes *SLC19A1* and *ABCB1*) in comparison with the MTX toxicity on plasma. They observed only 26% of reduction in MTX clearance of patients with 677TT genotype, although the effect of magnitude on MTX clearance was not quantified for these studies [28], but it suggests an absorption of MTX by leukemic cells and, consequently, low toxicity in plasma levels. In addition to the *1298A* > *C* polymorphism, they suggest it is also associated with lower risk of high-dose-MTX-associated leucopenia [28].

Unfortunately, it was not possible to examine all the genotyped patients relative to the *G80A* (*RFCh*) polymorphism. We show a small toxicity analysis in genotyped patients; however, the toxicity analysis (blood, kidney, and liver) suggests that the blood system has a toxicity degree more present among all the patients, presenting mainly leukopenia and thrombocytopenia. In the present study, we found that the *80AA* genotype, although it had the lowest MTX plasma level up to 24 hours, showed a small reduction of MTX plasma concentrations in the period of 24 to 48 hours. Thus, we can suggest that patients carrying the *80AA* genotype have a difficulty in metabolism of the chemotherapeutic to the transport the drug into the cell and, consequently, presentation of adverse effects. Chiusolo et al. [29] found in 54 patients with ALL a median age of 52 years (range 15–78 years); they found no influence of the *G80A* polymorphism on toxicity development and no correlation with MTX plasma levels (evaluated at 24 h and 48 h), but they point out a significant difference in overall survival rate according to genotypes; in fact, in the Kaplan–Meyer analysis, patients carrying the *80A* variant had a better prognosis than the patients with the *80GG* genotype, showing a better survival rate.

In our study, the patients with ALL-pediatric showed that the genotypic frequencies of the *C677T* polymorphism are similar to frequencies found in Egyptian, German and English populations, while the *A1298C* polymorphism showed frequency similar to Egyptian, French-Canadian, Italian, Japanese, and English populations [30].

Although there was no statistical significance for the *A1298C* polymorphism, the patients with mutant alleles (*677T* and *1298C*) showed a better survival, suggesting that these polymorphisms could be involved in a prognostic good for leukemia.

Regarding to the *G80A* (*RFCh*) polymorphism, we show that up to 80 months of treatment, the patients with *80GG* genotype had low survival. Chiusolo et al. [29] analyzed the overall survival of 49 patients, predominantly adult (15–78 years), noting a relation of worse survival in patients with *80GG* genotype, but they did not identify the MTX toxicity related to genotypes.

In studies of population genetic, they show that the allele frequency varies considerably among different ethnic and geographical areas [4]. Moreover, our population is considered heterogeneous, originated from African, Caucasian, and Native American ancestral individuals. Compared to the studies of Thirumaran et al. [31] (174 Italian patients) and Thirumaran et al. [31] (460 german patients), our study found no association statistically significant for the *A1298C* polymorphism; this association is not only a difficulty just present in multiethnic populations, but also is observed in German children [31] and Korean adults [32].

However, further studies should approach the treatment time and protocol, analyzing the risks of the polymorphisms and the folate route in different pediatric populations, because it would be important to conduct a meta-analysis study in order to get an appropriate treatment for the patients with polymorphisms unfavorable to the leukemia treatment.

## Figures and Tables

**Table 1:** Characteristics of the patients to ALL according to genotypes of the *C677T*, *A1298C,* and *G80A* polymorphisms.

	*n* = 126			*n* = 118			*n* = 118		
	677 CC (%)	677 CT (%)	677 TT (%)	1298 AA (%)	1298 AC (%)	1298 CC (%)	80 AA (%)	80 GA (%)	80GG (%)
Gender	71	46	9	50	43	25	42	51	25
Male	32 (25.4)	24 (19.0)	5 (04.0)	25 (21.2)	17 (14.5)	12 (10.1)	21 (17.8)	20 (17.0)	13 (11.0)
Female	39 (31.0)	22 (17.4)	4 (03.2)	25 (21.2)	26 (22.0)	13 (11.0)	21 (17.8)	31 (26.3)	12 (10.1)
Age									
<9 years	37 (29.4)	23 (18.2)	4 (03.2)	20 (17.0)	27 (22.9)	17 (14.5)	19 (16.1)	31 (26.3)	14 (11.8)
≥9 years	34 (27.0)	23 (18.2)	5 (04.0)	30 (25.4)	16 (13.5)	8 (06.7)	23 (19.5)	20 (17.0)	11 (09.3)
Living	52 (41.3)	34 (27.0)	7 (05.5)	40 (33.9)	30 (25.4)	19 (16.1)	31 (26.3)	42 (35.6)	18 (15.3)
Dead	19 (15.1)	12 (09.5)	2 (01.6)	10 (08.5)	13 (11.0)	6 (05.1)	11 (09.3)	9 (07.6)	7 (05.9)
Ratio (L/A)^‡^	0.36	0.35	0.28	0.25	0.43	0.31	0.35	0.21	0.39

^‡^ *P* value significant; L: living; D: dead.
